# Cartilage Conduction Is Characterized by Vibrations of the Cartilaginous Portion of the Ear Canal

**DOI:** 10.1371/journal.pone.0120135

**Published:** 2015-03-13

**Authors:** Tadashi Nishimura, Hiroshi Hosoi, Osamu Saito, Ryosuke Miyamae, Ryota Shimokura, Toshiaki Yamanaka, Tadashi Kitahara, Harry Levitt

**Affiliations:** 1 Department of Otolaryngology-Head and Neck surgery, Nara Medical University, Kashihara, Japan; 2 Nara Medical University, Kashihara, Japan; 3 The City University of New York, New York, New York, United States of America; University of South Florida, UNITED STATES

## Abstract

Cartilage conduction (CC) is a new form of sound transmission which is induced by a transducer being placed on the aural cartilage. Although the conventional forms of sound transmission to the cochlea are classified into air or bone conduction (AC or BC), previous study demonstrates that CC is not classified into AC or BC (Laryngoscope 124: 1214–1219). Next interesting issue is whether CC is a hybrid of AC and BC. Seven volunteers with normal hearing participated in this experiment. The threshold-shifts by water injection in the ear canal were measured. AC, BC, and CC thresholds at 0.5–4 kHz were measured in the 0%-, 40%-, and 80%-water injection conditions. In addition, CC thresholds were also measured for the 20%-, 60%-, 100%-, and overflowing-water injection conditions. The contributions of the vibrations of the cartilaginous portion were evaluated by the threshold-shifts. For AC and BC, the threshold-shifts by the water injection were 22.6–53.3 dB and within 14.9 dB at the frequency of 0.5–4 kHz, respectively. For CC, when the water was filled within the bony portion, the thresholds were elevated to the same degree as AC. When the water was additionally injected to reach the cartilaginous portion, the thresholds at 0.5 and 1 kHz dramatically decreased by 27.4 and 27.5 dB, respectively. In addition, despite blocking AC by the injected water, the CC thresholds in force level were remarkably lower than those for BC. The vibration of the cartilaginous portion contributes to the sound transmission, particularly in the low frequency range. Although the airborne sound is radiated into the ear canal in both BC and CC, the mechanism underlying its generation is different between them. CC generates airborne sound in the canal more efficiently than BC. The current findings suggest that CC is not a hybrid of AC and BC.

## Introduction

The conventional forms of sound transmission to the cochlea are classified into air or bone conduction (AC or BC). For AC, sound radiated from transducers to the air reaches the ear drum, and vibrates it. The transmission from the transducer to the ear drum is mediated by the air in the canal. In contrast, for BC, the delivered vibrations are transmitted to the cochlea via bone. The transducer is clinically placed on the skull bone such as the mastoid and forehead bone. In addition, if a transducer is attached to soft cranial tissue such as the fontanel, eye tissue, cheek, or neck, auditory sensations are also induced, which are referred to as non-osseous BC [1, 2, 3]. With regard to the transmission pathway, although BC involves various components such as sound radiated into the ear canal, middle ear ossicle inertia, inertia of the cochlear fluids, compression of the cochlear walls, and pressure transmission from the cerebrospinal fluid [4], the pathway via the ear drum is not the dominant pathway for BC in an open ear [5].

**Fig 1:**
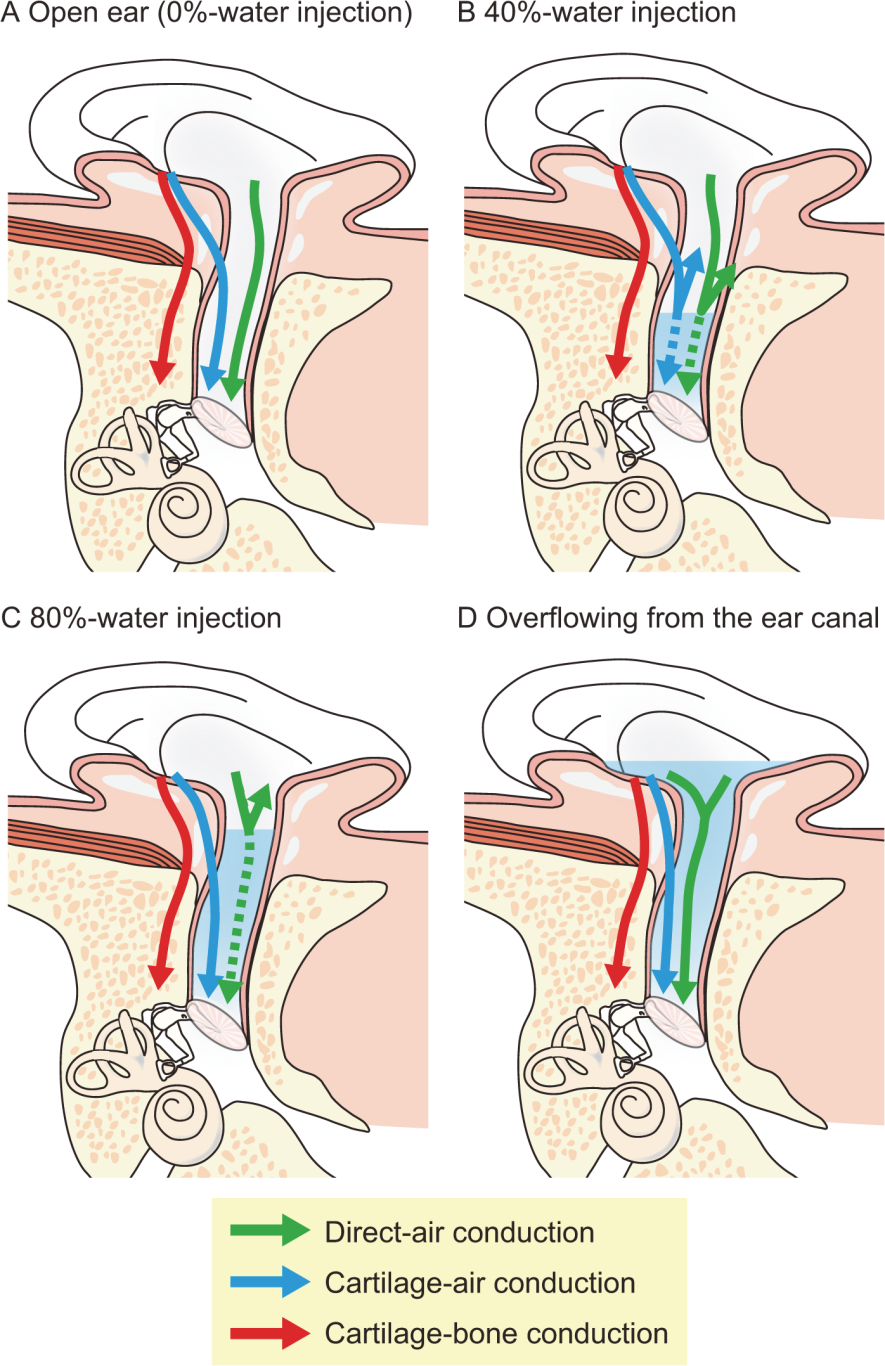
Sound transmission pathways in cartilage conduction. Part A shows three possible transmission pathways when the transducer is placed on the cavity of the concha [8, 9]. In the first pathway, vibrations of the transducer directly produces air-borne sound, some of which reaches the ear canal and is transmitted to the cochlea via the conventional pathway in air conduction (AC). This pathway is termed “Direct-AC”. In the second pathway, vibrations of the aural cartilage and soft tissue are transmitted to the cartilaginous portion. These vibrations induce an acoustic signal in the canal which is transmitted by AC to the eardrum. This pathway is termed “Cartilage-AC”. In the third pathway, vibrations of the aural cartilage and soft tissue are transmitted via the skull. This pathway is termed “Cartilage-bone conduction”. Part B, C, and D show the change in the sound transmission when the water is injected into the ear canal. When 40% of the ear canal is filled with water, the surface of the water probably levels at the bony potion. In this condition, the Direct-AC and Cartilage-AC are interrupted (Part B). When the water is additionally injected to 80% of the ear canal, the surface of the water probably reaches the cartilaginous portion. The vibration of the cartilaginous portion is efficiently transmitted to the eardrum, which is mediated by the injected water (Part C). Consequently, if the vibration of the cartilaginous portion contributes to the sound transmission, the threshold will first be elevated by the 40%-water injection, and be improved by the 80%-water injection. When the transducer touches the water (overflowing-water injection condition), the vibration is directly transmitted to the water (Part D).

Hosoi found that a clear sound can be heard when a vibration signal is delivered to the aural cartilage from a transducer [6]. Remarkable loudness change between touching on- and off-tragus conditions suggests the vibration of aural cartilage participates in sound transmission. This form of signal transmission is referred to as "cartilage conduction (CC)" [6, 7]. With regard to the sound transmission, the sound delivered to the aural cartilage can travel to the cochlea via three possible pathways as shown in Fig. 1A [8, 9]. In our previous study, the output levels at the thresholds with and without an earplug were compared among AC, BC, and CC [9]. Direct-AC is interrupted with the earplug inserted. In the results, the CC threshold increased by the insertion, implying the contribution of Direct-AC to the sound transmission. Without the earplug, the thresholds in force level for CC were lower than those for BC at all frequencies. Inserting the earplug, no increases in the CC thresholds were observed at the frequencies of 0.5 and 1 kHz. Even with the earplug, the CC thresholds below 2 kHz were lower than those of BC, implying the contribution of Cartilage-AC to the sound transmission. These findings suggested that the Direct-AC and Cartilage-AC, not Cartilage-BC, dominate the sound transmission. In another previous study of ours, the threshold with and without an earplug were compared for three conditions; the transducer being on the tragus, pretragus, and mastoid [10]. With the earplug inserted, the Direct-AC is interrupted. Although the thresholds were similar for all conditions, the thresholds for the tragus condition were most sensitive below 2 kHz. The previous study suggested that the direct vibration of the aural cartilage enhance sound transmission except at 4 kHz. While vibrations are delivered to body parts, the characteristics of CC are different from not only those of AC but also those of BC.

An important difference between BC and CC is the sound transduction and transmission. For BC, a heavy transducer of large mass is tightly pressed against the mastoid or forehead, which is needed to induce vibrations in the skull. In contrast, the aural cartilage is lighter, and can be vibrated with smaller energy than the skull. For CC, the vibrations in the skull are not always required, and a lightweight transducer is placed on the aural cartilage with a very small fixation force. Therefore, CC is more convenient and comfortable than BC.

One of the clinical applications of CC is utilized for hearing aids [7, 8, 11]. For instance, in the hearing disorder due to aural atresia, conventional AC hearing aids do not always provide a sufficient benefit. BC hearing aids are used in such cases as an alternative device. Unfortunately, BC hearing aids involve various problems derived from the fixation of the transducer. Long term use can also cause skin irritation, long-continued depressions in the skin, and discomfort [12]. On the other hand, hearing system utilizing CC may be a possible effective device. With regard to the sound transmission, in the cases of bony aural atresia, the efficacy of sound transmission for CC may be lower than that for BC because the CC transducer is not designed for vibrating the skull [8]. On the other hand, in the ear with fibrotic aural atresia, vibration of aural cartilage can be transmitted to the cochlea via the fibrotic tissue (fibrotic tissue pathway), if the fibrotic tissue is connected with ossicles. In our previous study, BC and CC thresholds were measured in the ear with fibrotic aural atresia [13]. In the ears with the presence of a fibrotic tissue pathway, the CC thresholds were lower than the BC thresholds at 0.5 and 1 kHz. Because the vibration of the skull is not required for this transmission, the energy of the vibration to be perceived may be lower. The benefit of CC hearing aid is therefore enhanced in the ear with fibrotic aural atresia. In practice, a great benefit of prototype CC hearing aid was observed in the patient with postoperative fibrotic aural atresia [7, 8].

Next interesting issue is whether CC is a hybrid of AC and BC. That is, there is a question whether the sound transmissions of CC in open and occluded ears are identical to those of AC and BC, respectively. If the vibration of the cartilaginous portion provided the characteristics of CC, Cartilage-AC and fibrotic tissue pathway would contribute to the sound transmission in an open ear and a fibrotic aural atresia, respectively. The comparison of the thresholds with and without an earplug demonstrated the contribution of Cartilage-AC [10]. However, the occlusion effect and the airborne sound from the earplug might influence the results. Shimokura et al. [14] measured the sound in the ear canal using a probe microphone with a transducer touching and non-touching the entrance of the ear canal. In the touching condition, the sound pressure level in the ear canal was an average of 25.5 dB higher than in the non-touching condition. These findings suggest that the vibration of the cartilaginous portion play an important role in CC. If the vibrations of the cartilaginous portion are evaluated, the results may establish the difference in sound transmission mechanism among AC, BC, and CC. In this study, threshold-shifts by water injection were measured for AC, BC, and CC. Fig. 1 illustrates the influence of water injection on the three components of CC. The ear canal consists of bony and cartilaginous portions. The medial half or two-thirds consists of the bony portion [15, 16, 17]. Accordingly, the water level for 40% water injection lies within the bony portion of the ear canal. When the water is filled within the bony portion, the Direct-AC and Cartilage-AC are interrupted (Fig. 1B). The water level for 80% water injection includes a substantial part of the cartilaginous portion of the ear canal. When the water reaches the cartilaginous portion, its vibration can be transmitted directly to the injected water (Fig. 1C). This route avoids the impedance mismatch between the air and water, and no occluded space exists. If the vibration of the cartilaginous portion dominates the perception, the thresholds will increase with the 40%-water injection, and then decrease by the additional injection. When the injected water overflows from the ear canal, the transducer directly vibrates the injected water without the mediation of the canal wall (Fig. 1D). If the threshold is improved only in this overflowing-water injection condition, it is suggested that the transmission through the cartilaginous portion is not dominant pathway. Thus, the relationship between the thresholds and injected water volume reveal the contribution of the three components.

## Materials and Methods

### Subjects

Seven volunteers (3 females and 4 males; 28–37 years old) with normal hearing participated in this experiment. The experimental procedure was approved by the ethics committee of Nara Medical University. Participants provided written informed consent before being enrolled. Before the experiment, AC and BC thresholds at frequencies of 0.5, 1, 2, and 4 kHz were obtained for each ear, and we confirmed that their thresholds were within 20 dB HL. For each subject, the ear with lower average BC threshold for the four test frequencies was selected for the study.

### Procedure

**Fig 2:**
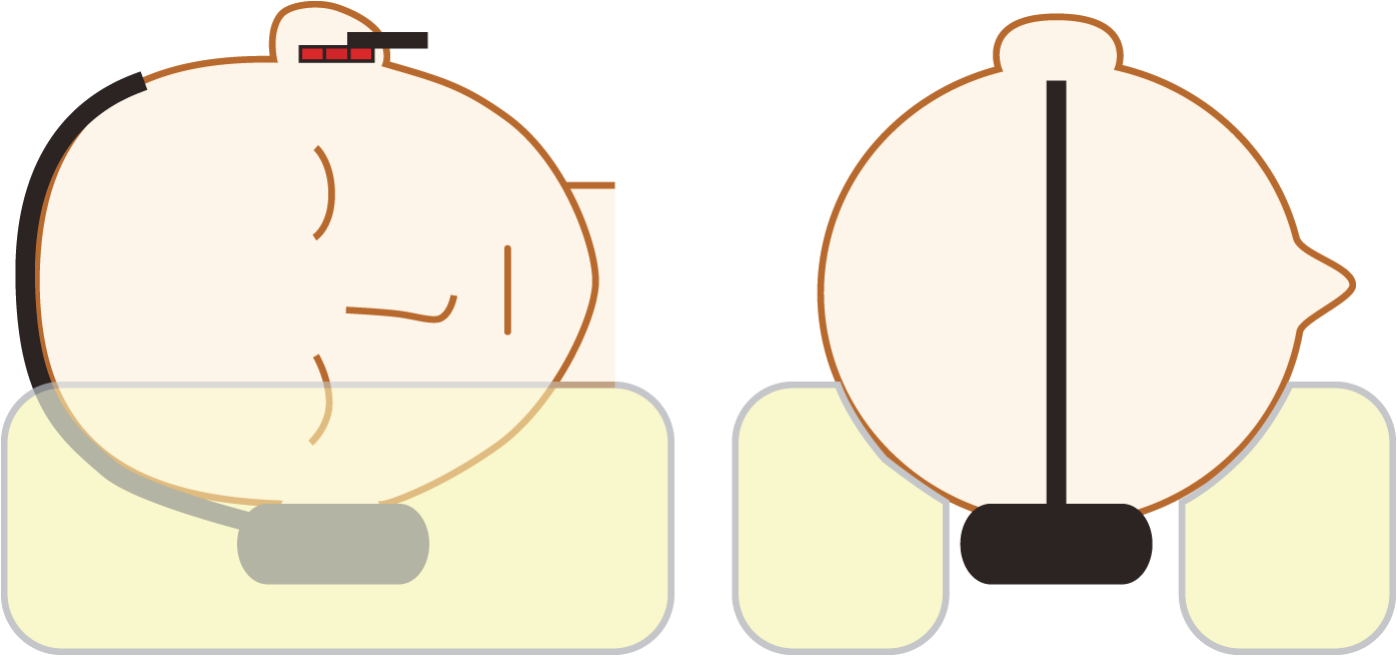
Subject’s head condition during the measurements. The subject’s head was fixed in the condition of the entrance of the ear canal facing up to the ceiling. The pillow had the space which enabled a masker earphone to be worn without the interference with the pillow.

The subject lay on a bed in a lateral recumbent position. The entrance of the ear canal faced up to the ceiling, and the head was fixed to avoid the water fluctuating in the canal (Fig. 2). The temperature of the injected water was set at 37 degrees Celsius to prevent vertigo. Prior to obtaining measurements, the volume of the ear canal for each subject was measured by injecting water into the ear canal, as described above. The volume of water to be injected was then determined for each experimental condition. The volume of water filling the ear canal but not overflowing into the cavity of the concha was defined as 100% full.

**Fig 3:**
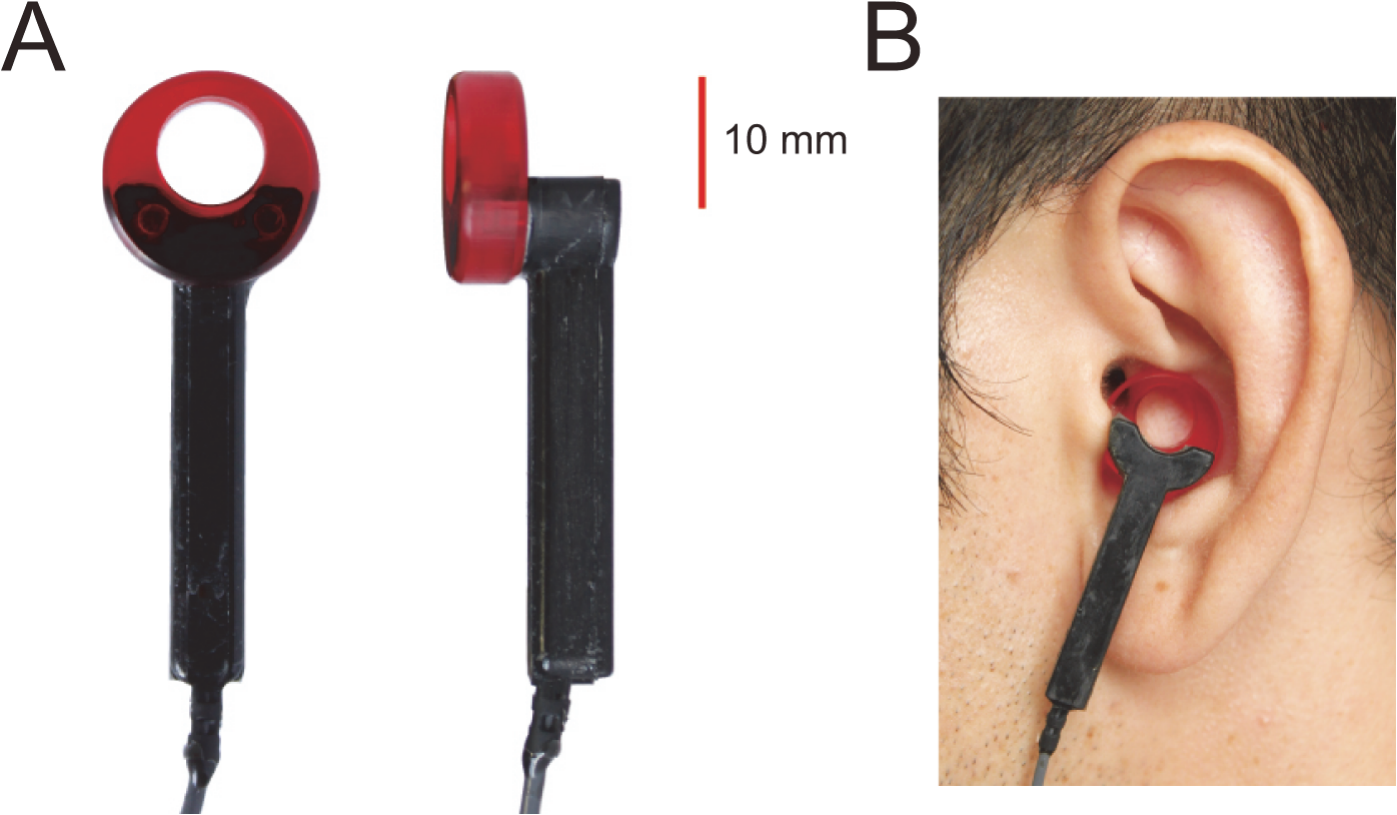
Cartilage conduction transducer. Part A and B show the cartilage conduction transducer and its fixation in the cavity of the concha.

The thresholds for AC, BC, and CC sound delivery were measured in the 0%-, 40%-, and 80%-water injection conditions in that order. In addition, CC thresholds were also measured for the 20%-, 60%-, 100%-, and overflowing-water injection conditions in order to obtain a more detailed evaluation of the relationship between amount of water injection and CC thresholds. For the 100%-water injection condition, we confined the transducer away from the injected water. After the measurement of the 100%-water injection condition, additional water was injected until half of the ring glued to the CC-transducer tip was soaked in the water (Fig. 3 shows the CC transducer and the attached ring). The four sets of measurement (AC, BC, and two CC series) were carried out in a randomly assigned order.

### Threshold measurement

The thresholds were measured using a transformed up-down procedure as described in the previous study [9]. A two-alternative forced-choice (2AFC) procedure with a decision rule that estimated the 70.7% positive response on the psychometric function was employed [18]. The stimulus frequency was set at 0.5, 1, 2, and 4 kHz, respectively. Tone bursts of 500 ms including rise/fall ramps of 10 ms were employed for the stimulus. The threshold was measured twice, and averaged. Narrow band masking noise was presented at the opposite ear, as shown in Fig. 2. The pillow under the listener’s head had a space large to allow a masker earphone to be mounted on the non-test ear. The intensity of the masking noise was set at the AC threshold level obtained in the preliminary measurement plus 30 dB. The experiment was performed in a sound proof room.

### Instruments

The testing procedure was programed using RPvdsEX ver. 6.2 (Tucker-Davis Technologies, Gainesville, FL, USA). The stimulus output and response input were processed using a real–time processor (RP2.1, Tucker-Davis Technologies). The AC signal was delivered by an earphone (AT-02, Rion, Tokyo, Japan) and the BC signal was delivered by a bone vibrator (BR-41, Rion). The bone vibrator was placed on the mastoid and its fixation force was approximately 5.4 N. These earphone and bone vibrator were calibrated with a sound pressure meter (AG-64; Rion) and artificial mastoid (Type 4930; Brüel & Kjær, Nærum, Denmark).

A lightweight bimorph piezoelectric transducer was used to deliver the CC signal. Fig. 3 provides several views of the CC transducer and how it is placed on the cavity of the concha, which was considered the most appropriate site for efficient sound transmission. A very small fixation force was used to attach the CC transducer to the aural cartilage. The CC transducer weighs 6 g and the fixation force excluding the stiffness of the conchal cartilage was estimated to be approximately 0.06 N. The properties of the transducer are described in a previous study [9]. The output level of the CC transducer was calibrated with the artificial mastoid (Type 4930). A standard fixation force of 5.4 N was used. A large fixation force is necessary for BC transducers [19, 20], but a much smaller fixation force is adequate for a CC transducer attached to the aural cartilage. Because the CC transducer was placed on the cavity of the concha with a small fixation force, the force level transmitted to the aural cartilage might be less than the value measured with the artificial mastoid.

### Statistical analysis

The threshold-shifts were calculated by subtracting the threshold at the 0%-water injection condition from that at each injection volume. The thresholds and threshold-shifts were compared among AC, BC, and CC. The data were analyzed using a three-way repeated-measures analysis of variance (ANOVA), with mode of conduction, frequency, and water volume as within-subject factors. A second analysis was performed on the CC data in which water injection was increased in steps of 20%. These data were analyzed using a two-way repeated-measures ANOVA, with frequency and water volume as within-subject factors. In order to evaluate the efficacy of the sound transmission without the Direct-AC component, the thresholds in force level were compared between BC and CC in the 80%-water injection condition. The data were analyzed using a two-way repeated-measures ANOVA, with mode of sound conduction and frequency as within-subject factors. These statistical analyses were performed by SPSS ver. 22 (International Business Machines Corporation, Armonk, New York). Bonferroni method was used for post-hoc comparisons. The significance level was set at 0.05.

## Results

With regard to the thresholds, a three–way repeated-measures ANOVA was performed on the measured thresholds. Statistically significant effects were observed for each of the main factors; mode of conduction (*F* [2, 12] = 551.31, *p* < 0.001), water volume (*F* [2, 12] = 582.71, *p* < 0.001), and frequency (*F* [3, 18] = 16.63, *p* < 0.001). All interactions among the main factors were significant (*p* < 0.001). For AC, water injection significantly elevated thresholds at all frequencies. Additional water injection from 40% to 80% significantly elevated AC thresholds even higher at 4 kHz. For BC, water injection significantly reduced the thresholds at 0.5 and 1 kHz, and significantly elevated thresholds at 2 and 4 kHz. No significant differences in BC thresholds were observed between the 40%- and 80%-water injection conditions. For CC, the thresholds were significantly elevated by the 40% water injection. However, additional water injection from 40% to 80% dramatically improved the thresholds at 0.5 and 1 kHz.

**Fig 4:**
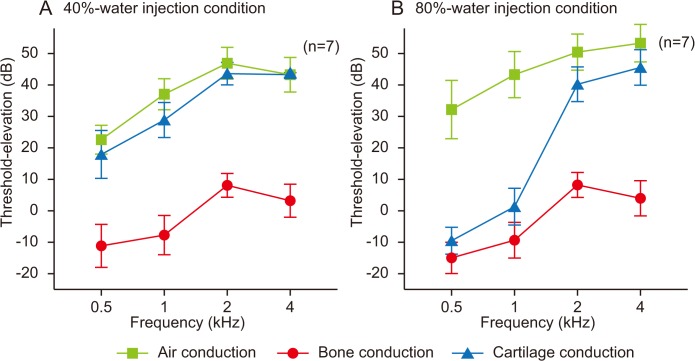
Threshold-shifts by the 40%- (A) and 80%- (B) water injections. Threshold-shifts from the 0%-water injection condition were described. The additional water injection from 40% to 80% of the ear canal remarkably improved the cartilage conduction thresholds at 0.5 and 1 kHz. Vertical bars indicate standard deviations.

The threshold-shifts resulting from the injection of water are shown in Fig. 4A and 4B. A three–way repeated-measures ANOVA revealed statistically significant effects for mode of conduction (*F* [2, 12] = 524.20, *p* < 0.001), water volume (*F* [1, 6] = 52.20, *p* < 0.001), and frequency (*F* [3, 18] = 123.94, *p* < 0.001). All interactions among these factors were significant (*p* < 0.001). No significant differences in threshold-shifts at 40% water injection were found between AC and CC. In the 80%-water injection condition, the threshold-shifts for CC at 0.5 and 4 kHz corresponded to those for BC and AC, respectively. As for threshold-shifts at 1 and 2 kHz, the largest was AC, followed in order by CC and BC.

**Fig 5:**
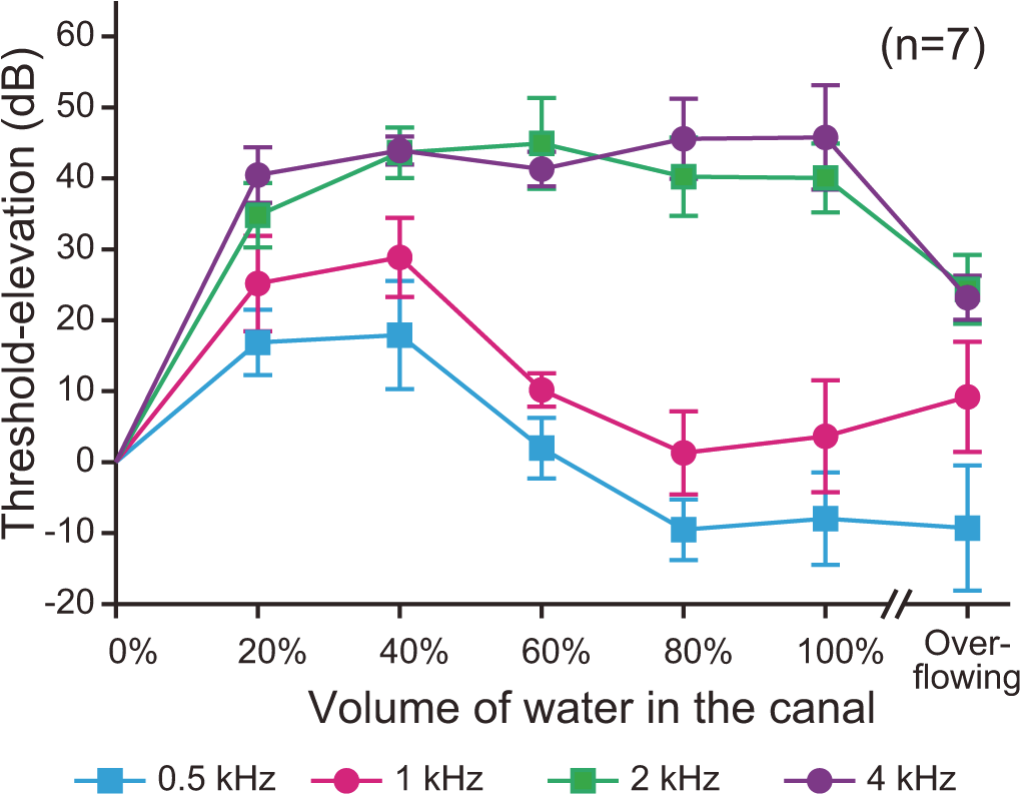
Threshold-shifts by the seven water-injection conditions in cartilage conduction. Threshold-shifts from the 0%-water injection condition were described. The water injection first elevated the thresholds at all frequencies. Beyond 60% of the ear canal, the thresholds at 0.5 and 1 kHz were improved. In addition, when the injected water overflowed to touch the transducer, the thresholds at 2 and 4 kHz were improved. Vertical bars indicate standard deviations.

Fig. 5 shows the threshold-shifts for the second set of data in which CC thresholds were obtained for water injected in steps of 20%. A two–way repeated-measures ANOVA revealed statistically effects for water volume (*F* [6, 36] = 155.47, *p* < 0.001) and frequency (*F* [3, 18] = 56.45, *p* < 0.001). The interaction between the main factors was significant (*F* [18, 108] = 47.15, *p* < 0.001). The CC thresholds were significantly elevated at all four frequencies by the initial 20% water injection. Increasing water injection from 40% to 60% significantly improved thresholds at 0.5 and 1 kHz. Thresholds at 2 and 4 kHz also significantly improved in the overflowing-water condition while the threshold-shift at 1 kHz significantly increased in the overflowing-water condition.

**Fig 6:**
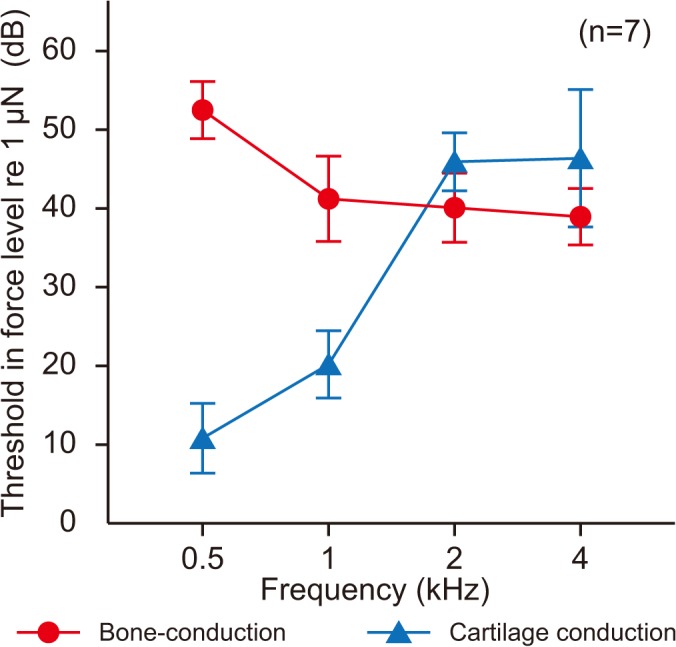
Thresholds in the 80%-water injection condition for bone and cartilage conductions. The thresholds were represented in force level referring to 1 μN. Vertical bars indicate standard deviations.

Fig. 6 shows the thresholds in force level (dB re 1 μN) for BC and CC in the 80%-water injection condition. A two–way repeated-measures ANOVA revealed statistically effects for stimulation condition (*F* [1, 6] = 80.56, *p* < 0.001) and frequency (*F* [3, 18] = 39.71, *p* < 0.001). The interaction between main factors was significant (*F* [3, 18] = 84.33, *p* < 0.001). The CC thresholds at 0.5 and 1 kHz were substantially lower than those for BC for the condition in which the water volume reached the cartilaginous portion of the ear canal. In contrast, the BC threshold at 2 kHz was significantly lower than that for CC. At the frequency of 4 kHz, the BC threshold was lower. However, no significance was observed between the BC and CC thresholds.

## Discussion

AC thresholds are elevated by more than 20 dB with 40% injection of water in the ear canal. The impedance mismatch between air and water is substantial and much of the AC signal reaching the air-water boundary is reflected back into the ear canal. There is an additional 5 to 10 dB elevation of the AC threshold (except at 4 kHz) for 80% water injection. This is presumably due to the longer water pathway that the signal has to travel through when the ear canal is almost full of water.

The BC thresholds at 2 and 4 kHz were elevated with the water injection. A possible explanation is that stray airborne sound from the BC transducer reaches the ear canal and is transmitted to the cochlea via the Direct-AC pathway where it improves the detectability of the signal reaching the cochlea by bone conduction. Stray airborne sound generated by a BC transducer can have sufficient power to lower a BC threshold [21, 22]. Another possible explanation is that water lying on the eardrum increased middle ear impedance by restricting the eardrum mobility and therefore, also, ossicular chain mobility. The change in the mobility can affect the BC thresholds. Because the threshold-shifts for BC were smaller than those for AC, the pathway via the skull is also important for high frequency sound transmission in BC. In contrast, BC thresholds at 0.5 and 1 kHz were lowered significantly by water injection even without the occlusion effect. The injected water can also mediate the transmission of the vibrations of the bony portion of the ear canal to the eardrum. This mediation by water in the ear canal can be accounted for by the reduced impedance mismatch between water and the bony ear canal wall compared to the larger impedance mismatch between air and bone.

Elevation of CC thresholds for 40% water injection was almost the same as for AC thresholds, implying that sound is transmitted via the eardrum in an open ear. This finding is in agreement with the previous suggestion that the Direct-AC or Cartilage-AC dominates sound transmission for sound delivered via a CC transducer [9]. It should also be noted that airborne sound generated by vibrations of the cartilaginous portion of the ear canal travel the same pathway to the cochlea as Direct-AC sound and that an air-water impedance mismatch in the ear canal will have an equal effect in impeding the flow of Direct-AC and Cartilage-AC sound in the ear canal. Thus, Cartilage-BC sound has no significant influence on the sound transmission in an open ear.

Additional water injection from 40% to 80% reduced the CC thresholds at 0.5 and 1 kHz substantially, implying that the impedance mismatch at the cartilage-water boundary is much smaller than that at the air-water boundary. Sound was transmitted with less reflection at the cartilage-water boundary, and more energy was conducted via the water, resulting better thresholds. Bear in mind that at 80% water injection the water level covers much of the cartilaginous portion of the ear canal. Vibrations of the cartilaginous portion of the ear canal are relatively intense at low frequencies but drop off at high frequencies because of the greater attenuation of sound in cartilage at high frequencies. Similar results were also observed in the measurement of the sound in the ear canal using a probe microphone [14]. In the touching condition, while the sound pressure level in the ear canal was an average of 25.5 dB higher than in the non-touching condition, remarkable increase was observed in the low frequency range. The average gain below 1 kHz was 35.1 dB, and the maximum gain was 45 dB at around 0.5 kHz. Furthermore, Shimokura et al. simulated the CC sound using a skull bone model attached with a model of aural cartilage (polyurethane resin pipe), and the sound pressure levels in the pipe below 1.5 kHz were close to measured values in ear canals from seven subjects [23]. Their results indicated that the vibration of the cartilaginous portion of the ear canal was the dominant factor for generating the sound pressure in the ear canal below 1.5 kHz. These findings suggest that the Cartilage-AC pathway dominates the CC thresholds at 0.5 and 1 kHz even in an open ear.

Airborne sound is generated in the ear canal not only for sound delivered by CC stimulation, but also for BC stimulation [4, 5]. Vibration of the skull contributes to sound transmission in BC, but not in CC [9]. If the airborne sound generated in BC is of the same intensity as in CC, the thresholds in force level for BC would be lower than those for CC. The remarkably low thresholds at 0.5 and 1 kHz for CC stimulation indicate that the cartilaginous portion of the ear canal vibrates more intensely to generate airborne sound in the canal more efficiently than for BC stimulation. The observation of no improvement with additional water injection from 40% to 80% for BC stimulation suggests that the vibration of the cartilaginous portion was not that intense. The BC transducer vibrates the skull primarily, and the cartilaginous portion of the ear canal may be vibrated as a consequence. In contrast, owing to the direct attachment of the CC transducer to the aural cartilage, the cartilaginous portion of the ear canal vibrates with much greater intensity relative to the bony portion. Although airborne sound is generated in the ear canal for both AC and BC stimulation, the mechanism underlying its generation in CC differs significantly from that in BC. Not only Direct-AC or Cartilage-BC but also Cartilage-AC which is not dominant component in AC or BC dominates the sound transmission. In addition, the airborne sound generated in CC is more intense than BC. These findings suggest that CC is not a hybrid of BC and AC but a unique form of sound delivery that has not been recognized.

An important advantage of CC is the transduction process. The lightweight transducer is attached with a fixation force that is substantially lower than that for BC. It is thus possible to have a CC transducer that is convenient and comfortable to wear for long periods. One possible application of CC is an alternative hearing device for aural atresia which cannot be treated appropriately with conventional AC or BC hearing aids [7, 8, 11]. A hearing aid employing CC is of particular benefit in an ear with fibrotic aural atresia [13]. An additional benefit is that the ear canal is not occluded by the transducer. As a consequence, the occlusion effect and the discomfort associated with long-term earphone use [12] is avoided. There are other possible applications of CC in hearing devices that offer new possibilities for the development of novel hearing aids, mobile phones, and open-ear transducers with unique significant features.

## References

[1] Sohmer H, Freeman S, Geal-Dor M, Adelman C, Savion I. Bone conduction experiments in humans—a fluid pathway from bone to ear. Hear Res. 2000;146: 81–88. 10913886 10.1016/s0378-5955(00)00099-x

[2] Watanabe T, Bertoli S, Probst R. Transmission pathways of vibratory stimulation as measured by subjective thresholds and distortion-product otoacoustic emissions. Ear Hear. 2008;29: 667–673. 10.1097/AUD.0b013e3181775dde 18596647

[3] Ito T, Röösli C, Kim CJ, Sim JH, Huber AM, Probst R. Bone conduction thresholds and skull vibration measured on the teeth during stimulation at different sites on the human head. Audiol Neurootol. 2011;16: 12–22. 10.1159/000314282 20453499

[4] Stenfelt S, Goode RL. Bone-conducted sound: physiological and clinical aspects. Otol Neurotol. 2005;26: 1245–1261. 16272952 10.1097/01.mao.0000187236.10842.d5

[5] Stenfelt S, Wild T, Hato N, Goode RL. Factors contributing to bone conduction: the outer ear. J Acoust Soc Am. 2003;113: 902–913. 12597184 10.1121/1.1534606

[6] Hosoi H. Approach in the use of cartilage conduction speaker. Japanese patent number 4541111, Filing date November 17, 2004.

[7] Hosoi H, Yanai S, Nishimura T, Sakaguchi T, Iwakura T, Yoshino K. Development of cartilage conduction hearing aid. Arch Mat Sci Eng. 2010;42: 104–110.

[8] Nishimura T, Hosoi H, Saito O, Miyamae R, Shimokura R, Matsui T, et al Benefit of a new hearing device utilizing cartilage conduction. Auris Nasus Larynx. 2013;40, 440–446. 10.1016/j.anl.2012.12.003 23395550

[9] Nishimura T, Hosoi H, Saito O, Miyamae R, Shimokura R, Matsui T, et al Is cartilage conduction classified into air or bone conduction? Laryngoscope. 2014;124:1214–1219. 10.1002/lary.24485 24166692

[10] Nishimura T, Hosoi H, Saito O, Miyamae R, Shimokura R, Matsui T, et al. Cartilage conduction efficiently generates airborne sound in the ear canal. Auris Nasus Larynx. 2014 Sep 5. pii: S0385-8146(14)00119-9. 10.1016/j.anl.2014.08.001 25199744

[11] Shimokura R, Hosoi H, Iwakura T, Nishimura T, Matsui T. Development of monaural and binaural behind-the-ear cartilage conduction hearing aids. Appl Acoust. 2013;74: 1234–1240.

[12] Dillon H. Hearing Aids. Stuttgart: Thieme; 2001.

[13] Morimoto C, Nishimura T, Hosoi H, Saito O, Fukuda F, Shimokura R, et al Sound transmission by cartilage conduction in ear with fibrotic aural atresia. J Rehabil Res Dev. 2014;51: 325–332. 10.1682/JRRD.2013.05.0128 24933730

[14] Shimokura R, Hosoi H, Nishimura T, Yamanaka T, Levitt H (2014) Cartilage conduction hearing. J Acoust Soc Am. 2014;135: 1959–1966. 10.1121/1.4868372 25234994

[15] Donaldson JA, Miller JM. Anatomy of the ear In: Paparella M.M. & Schumick D. A., editors, Otolaryngology, vol. 1 London: Saunders; 1980 pp. 26–31.

[16] Williams P, Roger W. The external acoustic meatus In: Gray’s Anatomy, London: Churchill Livingstone; 1980 pp. 1190–1193.

[17] Farrior JB. Incisions in tympanoplasty: anatomic considerations and indications. Laryngoscope. 1983;93: 75–89. 6337310 10.1288/00005537-198301000-00015

[18] Levitt H. Transformed up-down methods in psychoacoustics. J Acoust Soc Am. 1971;49: 467–477. 5541744

[19] Nilo ER. The relation of vibrator surface area and static application force to the vibrator-to-head coupling. J Speech Hear Res. 1968;11: 805–810. 5719235 10.1044/jshr.1104.805

[20] Lau CC. The effect of coupling force on bone conduction audiometry. Br J Audiol. 1986;20: 261–268. 3790771 10.3109/03005368609079025

[21] Lightfoot GR. Air-borne radiation from bone conduction transducers. Br J Audiol. 1979;13: 53–56. 486812 10.3109/03005367909078877

[22] Shipton MS, John AJ, Robinson DW. Air-radiated sound from bone vibration transducers and its implications for bone conduction audiometry. Br J Audiol. 1980;14: 86–99. 7417729 10.3109/03005368009078908

[23] Shimokura R, Hosoi H, Nishimura T, Iwakura T, Yamanaka T. (2014) Simulating cartilage conduction sound to estimate the sound pressure level in the external auditory canal. J Sound Vib. 2015;335: 261–268.

